# Why publish case reports in Physiological Reports

**DOI:** 10.14814/phy2.12784

**Published:** 2016-05-09

**Authors:** Thomas R. Kleyman, Julian R. E. Davis

**Affiliations:** ^1^ Departments of Medicine, Cell Biology, and Pharmacology and Chemical BiologyUniversity of PittsburghPittsburghPennsylvania; ^2^ Centre for Endocrinology and DiabetesFaculty of Medical and Human SciencesUniversity of ManchesterManchesterUK

Physiological Reports is an open access journal that is devoted to publishing important findings in all areas of physiology. In the field of medicine, physiology often has a key role in our understanding of both disease pathogenesis and therapeutics. This vital link between physiology and medicine has led the editors of Physiological Reports to launch a new initiative, publishing case reports that have a physiological focus. Case reports usually present what can be best described as anecdotal findings, and it is unusual for a case report to describe a truly novel finding or syndrome. Papers should be of interest to the physiology community because of the insights they offer regarding the nature of disease and its therapy. Authors will be discussing their findings within a broader context by analyzing and summarizing pertinent literature. Given the rapid advances in medical sciences, we believe that case reports will provide authors with the opportunity to review and synthesize newly published findings in a concise format that will provide our readers with new insights regarding disease pathogenesis and therapeutics. We particularly encourage submissions from physician trainees working in partnership with clinicians and physiologists. Our hope is that this initiative will enhance interactions between these groups.

